# Supplementary material for: The influence of parenting style and coping behavior on nonsuicidal self-injury behavior in different genders based on path analysis
Source: PeerJ. 2022 Dec 9;10:e14507. doi: 10.7717/peerj.14507 (PMC9745924; doi:10.7717/peerj.14507)
Supplement: Supplemental Information 2 [file peerj-10-14507-s002.docx]

Dear editors and readers, Our raw data tables adhere to the following rules: variables [behavior or phenomenon (marker number)], such as gender [male (1) and female (2)] indicate that gender is marked as 1 for male and 2 for female.

**Basic situation**

The following parameters in the basic status questionnaire were included: age [11-13(1), 14-16(2)], gender[male(1) and female(2)], physical status [weak(1), average(2), good(3)], academic performance [poor(1), average(2), good(3), excellent(4)], peer relationship [poor(1), average(2), close(3)], parental relationship [separation/divorce(1), domestic violence(2), quarrel conflict(3), harmony(4)], per capita monthly income of the family [< 3000(1), 3000-6000(2), 6000-10000(3), > 10000(4)], per capita housing area of the family [< 20(1), 20-40(2), 40-60(3), > 60(4)], parents' investment in their children [very low(1), low(2), average(3), high(4)], parents' expectations for their children [none(1), independent life(2), range oneself(3), attend university(4)], fathers' and mothers' education level [primary school(1), junior middle school(2), senior high school(3), university and above(4)].

**Coping style**

We used a middle school students' Coping Styles Scale to measure the subjects' coping styles in the face of events. There are 36 items on the survey scale, and each item is graded according to the four-level scoring standard of adoption, occasional adoption, occasional adoption, and frequent adoption. The subscale consists of two parts: (1) problem-oriented coping subscale, which includes three factors: P1 problem-solving, P2 seeking social support, and P3 positive rationalization explanation; (2) emotional-oriented coping subscale, which includes four factors: E1 avoidance, E2 escape, E3 venting, and E4 fantasy denial.

Each factor is composed of several items. The items are rated as 4 levels, [not adopted(1), occasionally adopted(2), sometimes adopted(3), frequently adopted(4)]. The factor scores can be added by the item scores, and the factor scores belonging to the same subscale are added together, that is, the subscale scores. The composition of each factor of the final subscale is as follows:

**problem-oriented coping subscale:**

(1) Problem solving: 1, 6, 11, 16, 22, 28, 33

(2) Seeking social support: 2, 7, 12, 17, 23, 29, 34

(3) Positive rationalized explanations: 3, 8, 13, 18, 24

**emotional-oriented coping subscale:**

(1) Patience: 19, 25, 30, 35

(2) Escape: 4, 9, 14, 31

(3) Vent emotions: 20, 26, 32, 36

(4) Fantasy, denial: 5, 10, 15, 21, 27

**NSSI**

NSSI behavior is defined according to DSM-5 diagnostic criteria, and the following true-or-false questions are designed: (1) In the past year, there have been more than 5 acts of intentional injury to oneself that are not to end life and cause mild or moderate physical injuries such as bleeding, contusion, and pain; (2) The above behaviors have the purpose of extricating from a tense feeling or cognitive state and/or solving interpersonal difficulties and/or inducing a positive emotional state. Answering "yes" to the above two questions indicates that there is NSSI behavior. The conversion rules in the table are as follows:[No NSSI(0), NSSI(1)].

**Parenting Approaches Evaluation Scale**

The parenting styles evaluation scale (EMBU) was introduced to effectively evaluate the relationship between parenting style and individual NSSI behavior. The scale contains 66 items, of which (1) the subscale of paternal rearing style contains 6 factors, which are F1 emotional warmth and understanding, F2 severe punishment, F3 refusal and denial, F4 preference for subjects, F5 excessive interference, and F6 excessive protection; (2) The subscale of maternal rearing style includes five factors: M1 emotional warmth and understanding, M2 severe punishment, M3 refusal and denial, M4 preference for subjects, and M5 excessive interference and protection.

The items of each factor of the father and mother scale are as follows:

**F1:**

(father's emotional warmth and understanding and care) :

2, 4, 6, 7, 9, 15, 20, 25, 29, 30, 31, 32, 33, 37, 42, 54, 60, 61, 66

**M1:**

(mother's emotional warmth and understanding and care):

2, 4, 6, 7, 9, 15, 25, 29, 30, 31, 32, 33, 37, 42, 44, 54, 60, 61, 63

**F2:**

(severe punishment of father):

5, 13, 17, 18, 43, 49, 51, 52, 53, 55, 58, 62

**M2:**

(severe punishment of mother):

13, 17, 43, 51, 52, 53, 55, 58, 62

**F3:**

(father's refusal and denial):

21, 23, 28, 34, 35, 45

**M3:**

(mother refused to deny)：

23, 26, 28, 34, 38, 39, 45, 47

**F4:**

(father prefers subjects):

3, 8, 22, 64, 65

**M4:**

(mothers prefer subjects):

3, 8, 22, 64, 65

**F5:**

(father's excessive interference):

1, 10, 11, 14, 27, 36, 48, 5, 56, 57

**M5:**

(excessive intervention of mother) :

1, 11, 12, 14, 16, 19, 24, 27, 35, 36, 41, 48, 50, 56, 57, 59

**F6:**

(father overprotective):

12, 16, 39, 40, 59

Note: among the 66 items, the father scale does not contain 19, 24, 26, 38, 41, 47, 54, 63; The mother scale does not contain 5, 10, 18, 20, 21, 40, 49, 66. For convenience, we asked subjects to answer all questions, but the above items were not included in the calculation and analysis.

The conversion rules in the table are as follows:[never (1), occasionally (2), frequently (3), always (4)]. (item 20, 50, and 56 are reverse scoring, i.e. "always" counts 1 point and "never" counts 4 points).
